# Digital Transformation and Disruption of the Health Care Sector: Internet-Based Observational Study

**DOI:** 10.2196/jmir.9498

**Published:** 2018-03-27

**Authors:** Maximilian Herrmann, Philip Boehme, Thomas Mondritzki, Jan P Ehlers, Stylianos Kavadias, Hubert Truebel

**Affiliations:** ^1^ Didactics and Educational Research in Health Science Faculty of Health Witten/Herdecke University Witten Germany; ^2^ Cardiovascular Research Bayer Aktiengesellschaft Wuppertal Germany; ^3^ Judge Business School University of Cambridge Cambridge United Kingdom; ^4^ Faculty of Health Witten/Herdecke University Witten Germany

**Keywords:** digital transformation, health care sector, health care reform, incremental innovation, disruptive innovation, organizational innovation, entrepreneurship, efficiency, models, organizational, diffusion of innovation, delivery of health care

## Abstract

**Background:**

Digital innovation, introduced across many industries, is a strong force of transformation. Some industries have seen faster transformation, whereas the health care sector only recently came into focus. A context where digital corporations move into health care, payers strive to keep rising costs at bay, and longer-living patients desire continuously improved quality of care points to a digital and value-based transformation with drastic implications for the health care sector.

**Objective:**

We tried to operationalize the discussion within the health care sector around digital and disruptive innovation to identify what type of technological enablers, business models, and value networks seem to be emerging from different groups of innovators with respect to their digital transformational efforts.

**Methods:**

From the Forbes 2000 and CBinsights databases, we identified 100 leading technology, life science, and start-up companies active in the health care sector. Further analysis identified projects from these companies within a digital context that were subsequently evaluated using the following criteria: delivery of patient value, presence of a comprehensive and distinctive underlying business model, solutions provided, and customer needs addressed.

**Results:**

Our methodological approach recorded more than 400 projects and collaborations. We identified patterns that show established corporations rely more on incremental innovation that supports their current business models, while start-ups engage their flexibility to explore new market segments with notable transformations of established business models. Thereby, start-ups offer higher promises of disruptive innovation. Additionally, start-ups offer more diversified value propositions addressing broader areas of the health care sector.

**Conclusions:**

Digital transformation is an opportunity to accelerate health care performance by lowering cost and improving quality of care. At an economic scale, business models can be strengthened and disruptive innovation models enabled. Corporations should look for collaborations with start-up companies to keep investment costs at bay and off the balance sheet. At the same time, the regulatory knowledge of established corporations might help start-ups to kick off digital disruption in the health care sector.

## Introduction

Digital transformation and disruptive innovation describe the comprehensive reorientation of an industry including its business models due to the coming of age of digital technologies: the digitization of products, services, and processes [1-4]. It is expected that digital transformation of the health care sector will be as disruptive as that seen already in other industries [1-3,5]. Despite new technologies being constantly introduced, this change has yet to materialize [6-9].

According to Christensen [10,11], disruptive innovation requires 3 elements: (1) a technological enabler that simplifies previously complicated tasks, (2) a business model innovation that profitably delivers these simplified tasks in an affordable and convenient way, and (3) a new value network that reinforces a stakeholder position in this ecosystem. Given these conditions, it becomes intuitive that often enough disruptors come from outside an industry (with a total rethinking of the current business practices). They encroach the existing market dominance of established players from the bottom up (ie, from segments and products or services that can be viewed as lower margin and perhaps less valuable for the incumbent corporations in the industry) [9]. The counterpart to disruptive innovation is incremental innovation, the improvement or enhancement of product features and services that already exist in a market [6,12,13].

Health care systems face major challenges with rising costs, increasing demand for provision of care in aging societies, and outcome problems [14,15]. It has been shown in the United States that despite the availabilty of high-tech medicine, the average standard of care remains low compared to its cost [14], and this phenomenon can also be seen on a global scale [9,14,16].

Recent examples show that digital technology can mitigate or even eliminate these challenges, thus improving health care delivery [17-20]. Despite all the hype of “digital,” why is the digital transformation of the health care sector still to be seen? One hurdle could be the heavily regulated nature of the sector. On one hand, regulations ensure that products reach the market with adequate safety, quality, and efficacy; on the other, regulating a complex industry could cause an innovation straightjacket because it is hard to predict the feasibility of innovative approaches well in advance [9,14,21,22]. For many patients, for whom health care remains expensive and at times inaccessible, the digital transformation offers the promise of better and cheaper care [6,10].

This study aims to provide an up-to-date comprehensive analysis of the transformational forces within the health care sector by looking at different stakeholders (life sciences, technology, and start-up companies). We evaluate their strategies on digital offerings and identify those that are disruptive or more incremental. We also point toward strategies that could enable digital disruption within the health care system.

## Methods

### Data

A systematic analysis was performed to screen for different technology and life science corporations regarding their digital transformation activities in the health care sector using 2017 Forbes 2000 data [23] from an annual ranking of the top 2000 companies in the world. The search terms “digital health,” “digital medicine,” “eHealth,” “health care,” “mHealth,” “outcomes-based reimbursement,” and “value-based care” were used to identify the 100 leading corporations. In addition, the 100 most successful start-up ventures active in the health care sector were identified based on the amount of funding they received as recorded from 2017 data by CBinsights [24]. We defined these efforts as “projects”.

### Evaluation of Identified Projects: Business Models, Solutions Provided, and Customer Needs Addressed

An expert panel consisting of 10 members with multiprofessional backgrounds in medicine, pharma, and economics rated these projects according to the following criteria [25,26]:

- Customer value proposition can be identified.
- Key resources can be identified.
- Key processes can be identified.
- Profit formula can be identified.

Each criterion was ranked from 0 (customer value proposition not given or not clear from the available sources) to 4 (customer value proposition can be readily identified). The last 3 criteria specifically allowed an assessment of the underlying business model [25,26]. The sums of scores from the 4 criteria were used to rank the projects and further look into the 20 highest ranked projects per group of companies (60 projects in total) in greater detail with regard to their regional location, customer value proposition, and solutions provided in connection with service, software, hardware, or platform to define different categories. These 60 projects were then evaluated according to 6 customer needs: adherence, diagnostic, lifestyle, patient engagement, prevention, or treatment. These categories were created by identifying similarities between the different projects and grouping them by which customer needs they addressed. The groups were named accordingly. A chi-square test was performed for the different companies to verify whether their provided solutions or their addressed customer needs come from the same distribution or are significantly different. All tests were performed with statistical significance of *P*<.05.

## Results

### Regional Distribution

More than 400 projects (Multimedia Appendix 1) were identified from the 100 leading start-up, life science, and technology companies. In our analysis of the 60 highest rated projects (Multimedia Appendix 2) identified by our expert panel, a high regional concentration with 40 out of 60 projects (66%) located in the United States was found. There was an aggregation of projects on the West Coast of the United States (Multimedia Appendix 3).

### Business Models, Solutions Provided, and Customer Needs Addressed

Multimedia Appendix 4 shows the results of our in-depth analysis of the customer value proposition and the underlying business model. Within the group of highest ranked projects, device-developing start-up projects assume a primary position. The second grouping represents different start-up projects that are less well-defined with respect to their customer value proposition and business model. In the third grouping, one can see that corporations from the technology and life science sectors appear to be more active in collaborations and in efforts to engage with topical experts in their targeted digital innovations.

### Provided Solutions

**Figure 1:**
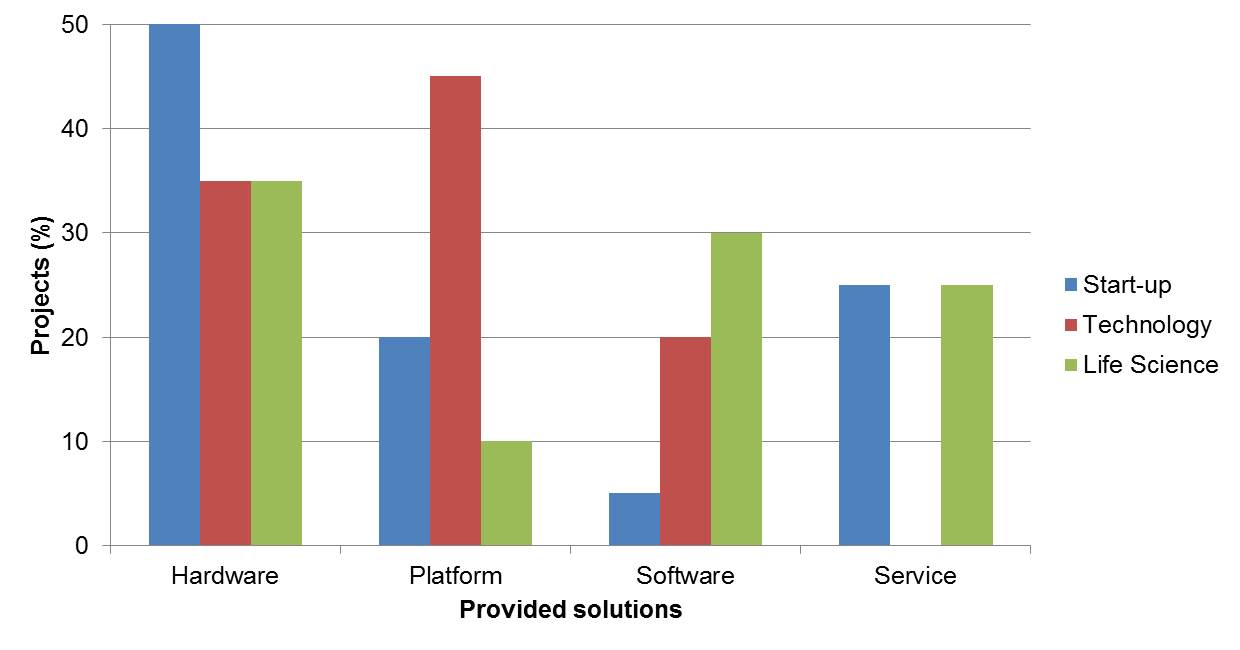
Distribution of identified types of solutions among the projects of the 3 industry players.

The analysis led to 4 distinct types of solutions that could be identified, as shown in Figure 1. All 3 industry players are engaging in projects that are represented across the different types of solutions. Specifically, while hardware solutions appear to be evenly distributed, projects on new services seem to be undertaken only by start-ups and life sciences companies. Interestingly we found a strong engagement of technology corporations in the platform field. Chi-square tests indicated there is no statistical evidence that start-up and technology solutions (χ²_3_=21.2, *P*<.001,), start-up and life science solutions (χ²_3_=26.9, *P*<.001), or technology and life science solutions (χ²_3_=30.2, *P*<.001) come from the same distribution. This indicates with respect to the structure of the solutions that the 3 groups have been pursuing distinctively different strategies.

### Customer Needs Addressed

**Figure 2:**
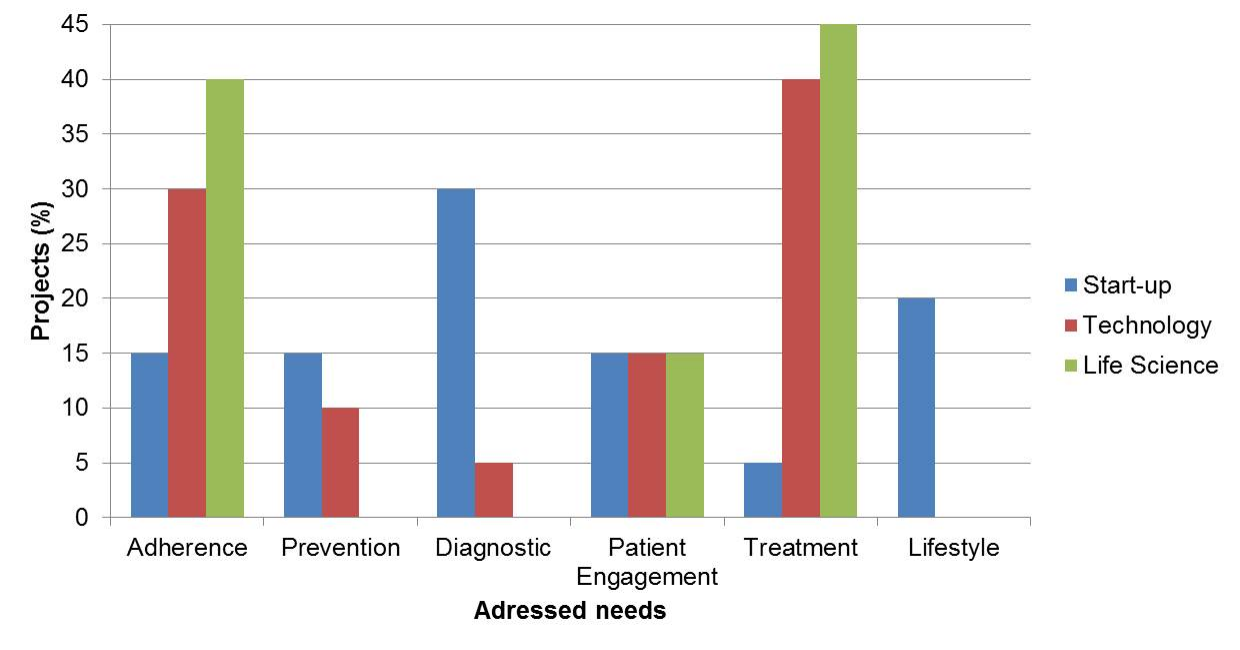
Distribution of customer needs addressed among the projects of the 3 industry players.

To analyze whether these projects were addressing similar or different customer needs, we focused on 6 customer needs that were further investigated: adherence, diagnostic, lifestyle, patient engagement, prevention, and treatment. As shown in Figure 2, distributions among the different companies’ foci were found. It could be shown that the start-up projects represent all patient needs consistently. Efforts from the life science sector were focused primarily on adherence- and treatment-related projects, while no major actions appeared for prevention, diagnostic, and lifestyle. Technology corporations were similar with no projects in the lifestyle field. Chi-square tests indicated there is no statistical evidence that start-up– and technology-addressed needs (χ²_5_=60.5, *P*<.001) or start-up– and life science–addressed needs (χ²_5_=85.3, *P*<.001) come from the same distribution. However chi-square tests indicated there is statistical evidence that technology- and life science–addressed needs come from the same distribution (χ²_4_=3.8, *P*=.435). That means that start-up companies have a significantly different focus than technology and life sciences corporations. However, the technology and life sciences corporations do not have statistically significant foci from each other.

### Intercategorial View

**Figure 3:**
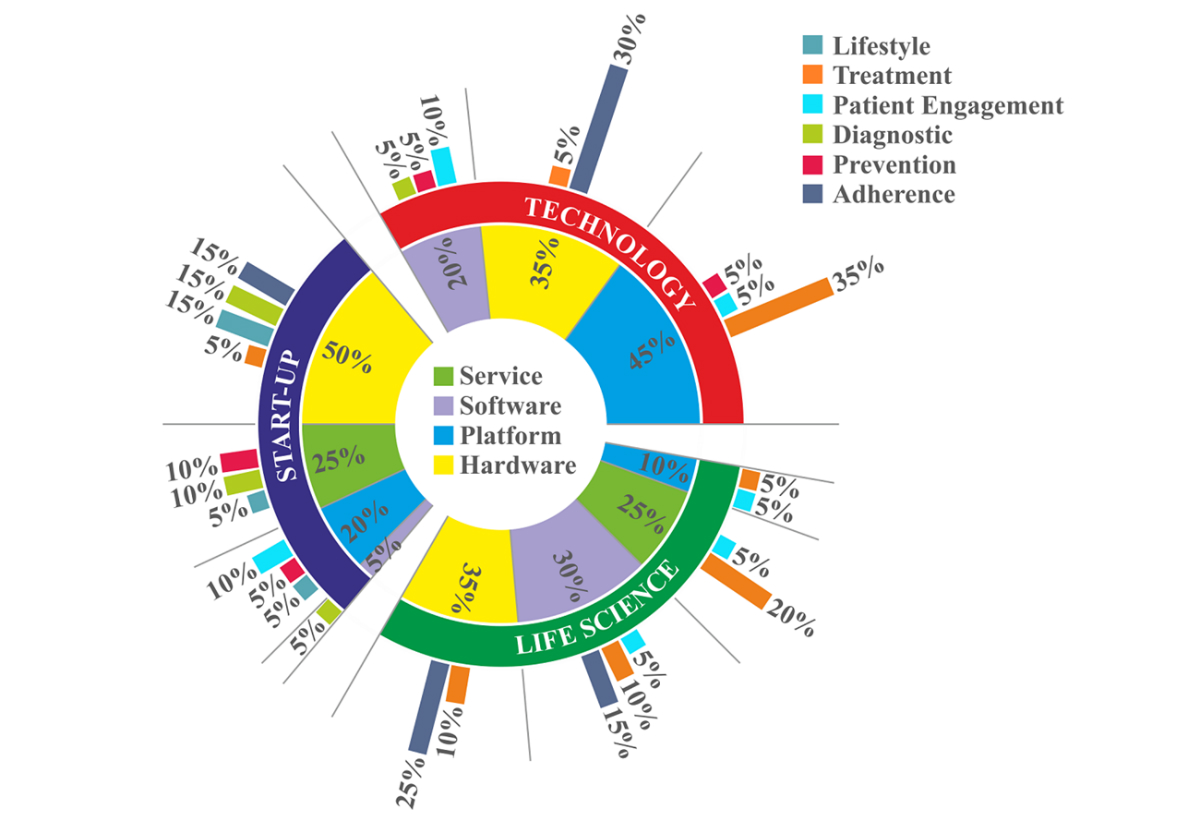
Solutions provided and needs addressed by category.

Combining the information from the previous analyses shows that adherence and treatment projects are preferred by corporations in the life science and technology sectors (Figure 3). Both seem to neglect lifestyle-focused projects. More importantly, start-up companies show a much broader focus in their efforts to address patient needs.

## Discussion

### Summary

The goal of the study was to provide an up-to-date comprehensive analysis of the transformational forces within the health care sector by looking at different stakeholders. Our results identified patterns showing that established corporations rely more on incremental innovation that supports their current business model, while start-up companies engage their flexibility to explore new market segments with notable transformations of established business models.

**Example of the focus of a life science company.**

Sanofi is a French-based life science company that has signed a value-based pricing contract with the US health insurer Cigna on a new cholesterol-lowering drug. The price is linked to patient cholesterol levels. If the drug fails to decrease the level of cholesterol as seen in clinical trials, Sanofi must further discount the drug.

**Example of a not fully developed value proposition and business model.**

xbird is a German-based start-up company that extracts millions of data points from smartphone sensors, wearables, and medical devices, combining environmental and digital biomarkers. Data scientists and medical experts combine these data and identify patterns leading to critical health events. This technology enables connection of adverse health events with behavioral causes and creates actionable insights for both doctors and patients. The company implements technology, new business models, and value propositions to use the collected data to avoid critical health events before occurrence.

### Regional Diversity

The data show a dominant positioning of projects in the United States. One reason could be that the United States shows the highest digital innovation potential through the provision of an extraordinary environment for disruptive innovation. Here our analysis indicates that 17 projects out of the 40 (43%) were located in California, and 10 out of the 17 projects (59%) are based in San Francisco and Mountain View, the heart of Silicon Valley. There might also be an increased interest for disruptive innovation in the health care sector in the United States due to its low efficiency [10].

### Principal Findings

The distribution of business models provided by the different projects indicates 3 main areas that distinguish large, established corporations (life science and technology) from start-ups. The younger start-up competitors appear to pursue solo efforts instead of collaborative endeavors and undertake efforts directed in spaces that are not pursued by others. When exploring further into the detailed solutions (Figure 1) and addressed needs (Figure 2), we find that start-ups display a wider approach toward the digital health care sector. This is contrasted by the more established life science and technology corporations which focus on adherence and treatment projects (Textbox 1). These supplement their existing market offerings, and therefore could be viewed as initial departures from the existing business models. However, statistical results indicate that not a single cohort of companies has figured out what the right digital approach is and this reflects well in the traditional fermentation/converging period that many industries exhibit during large shifts in how business takes place.

With respect to the customer needs addressed, we notice an interesting effect. Technology and life science corporations seem to address similar customer needs. Either the technology companies have not been really creative in addressing the digital challenges or the technology companies were creative in the beginning but the life science sector has caught up pretty fast. The start-up endeavors are significantly different than all others, which indicates that disruption would come from the start-ups because they are playing in different domains than established corporations. In that regard, they exhibit strong differentiation in both the supply and the demand side from a digital innovation standpoint. These observations can echo the underlying structural elements in the disruptive innovation theory of Christensen [10,11]. In that light, incumbent corporations within the life science sector (eg, pharmaceutical corporations) tend to work on more effective drugs, but they lack the capability to directly interact with the patient and therefore transform their competitive position by additional (recently termed “beyond the pill”) offerings. Aligned with this expectation, we identified that their projects show a clear tendency to offer digitally enhanced outputs but such efforts tend to be incremental innovations that stick to traditional market strategies. Interestingly, providers of consumer care products like Fitbit and Jawbone are further penetrating the health care sector, moving beyond lifestyle products for customers interested in health self-monitoring and toward offerings that compete directly with more established health care corporations like Medtronic.

Start-up offerings of products and services appear in some cases not fully developed with respect to the exact value proposition of the offering (Multimedia Appendix 4) and business model (Textbox 2).

In general, start-ups use their flexible structures to pursue radically new avenues with the help of novel technologies, business models, and value networks that provide disruptive solutions to a wide variation of customer and patient challenges. Within our sample, established technology and life science corporations aim through their projects to address challenges that relate, to a significant extent, to the adherence and treatment dimensions of the customer value. Thus, they seem to be underrepresented in the remaining types of customer value. This offers evidence that established corporations focus on digital improvements of their existing business offerings and value proposition, which in turn signifies lesser interventions to their current business models. Their focus stands in sharp contrast to the diversity of start-ups, which seem to address diversified customer needs.

### Limitations

Our analysis, as the first capturing the phenomenon of disruptive innovation within the health care sector, has a few limitations. It is based on information available in the public domain, which might not allow for a comprehensive picture since some start-ups might overreport to attract funding and other start-ups might not have yet made a public splash, as they are rather early in their development process. At the same time, established large corporations for privacy reasons might underreport on their digital initiatives. The ratings by 10 evaluators might also blur stronger differences given their diverse educational backgrounds. Public domain data might lack the depth of information needed to allow for precise rating of the different characteristics assessed.

### Conclusion

Notwithstanding the limitations of our analysis, the emerging pattern allows us to differentiate innovating corporations within the health care sector with respect to their strategies in the context of the digital transformation in health care. Established corporations show strength in improving the business model dimensions they have been pursuing for a long time. Start-up companies appear more agile and able to make better use of radical new technologies and different business models moving toward new forms of disruptive innovations. Since the health care sector is tightly regulated, established players with an in-depth understanding of its regulatory mechanism might have clear advantages here, but start-ups are tackling this specific challenge well.

Start-ups with their agile culture and established technology or life science corporations with their regulatory knowledge might join strategic forces to drive the digital transformation of the health care sector. By engaging in collaborative efforts, corporations can keep costs at bay, while addressing all patient needs and claiming the investments off their balance sheet. Being in position to quickly adapt when a disruptive business model emerges will be the key for future revenues. A disruptive threat for both life science and start-up is the strong focus of technology corporations to establish platform business models and assume the necessary bargaining power to appropriate the value created. It remains to be seen whether the future market leaders of a transformed health care sector will be the existing corporations and current market leaders or new players who are going to emerge from the ranks of today’s start-ups.

## Appendix

Multimedia Appendix 1 List of identified projects.

Multimedia Appendix 2 The 60 highest ranked projects by expert evaluation.

Multimedia Appendix 3 Regional diversity of the 60 highest ranked projects.

Multimedia Appendix 4 Distribution of projects.

## References

[1] (2014). Harvard Business Review.

[2] Kreutzer R, Schallmo D, Rusnjak A, Anzengruber J, Werani T, Junger M (2017). Treiber und Hintergrunde der digitalen Transformation. Digitale Transformation von Geschäftsmodellen: Grundlagen, Instrumente und Best Practices.

[3] Valenduc G, Vendramin P (2017). Digitalisation, between disruption and evolution. Transfer Eur Rev Labour Res.

[4] Loucks J, Macaulay J, Norohna A, Wade M (2016). Digital Vortex: How Today's Market Leaders Can Beat Disruptive Competitors at Their Own Game.

[5] Klewes J, Popp D, Rost-Hein M (2016). Out-thinking Organizational Communications: The Impact of Digital Transformation.

[6] Hwang J, Christensen CM (2008). Disruptive innovation in health care delivery: a framework for business-model innovation. Health Aff (Millwood).

[7] Perakslis ED (2017). Strategies for delivering value from digital technology transformation. Nat Rev Drug Discov.

[8] Westerman G, Tannou M, Bonnet D, Ferraris P, McAfee A The digital advantage: how digital leaders outperform their peers in every industry.

[9] Porter ME, Teisberg EO (2004). Redefining competition in health care. Harv Bus Rev.

[10] Christensen C, Grossman J, Hwang M (2008). The Innovator's Prescription: A Disruptive Solution for Health Care.

[11] Christensen C (2013). The Innovator's Dilemma: When New Technologies Cause Great Firms to Fail.

[12] Hacklin F, Raurich V, Marxt C (2004). How incremental innovation becomes disruptive: the case of technology convergence.

[13] Assink M (2006). Inhibitors of disruptive innovation capability: a conceptual model. Euro J Innov Managem.

[14] Porter M, Teisberg E (2006). Redefining Health Care: Creating Value-Based Competition on Results.

[15] Christensen CM, Bohmer R, Kenagy J (2000). Will disruptive innovations cure health care?. Harv Bus Rev.

[16] Kohn LT, Corrigan JM, Donaldson MS (2000). To Err is Human: Building a Safer Health System.

[17] Labovitz DL, Shafner L, Reyes GM, Virmani D, Hanina A (2017). Using artificial intelligence to reduce the risk of nonadherence in patients on anticoagulation therapy. Stroke.

[18] McGrail KM, Ahuja MA, Leaver CA (2017). Virtual visits and patient-centered care: results of a patient survey and observational study. J Med Internet Res.

[19] Steinhubl SR, Muse ED, Topol EJ (2013). Can mobile health technologies transform health care?. JAMA.

[20] Kaufman N, Salahi A (2017). Using digital health technology to prevent and treat diabetes. Diabetes Technol Ther.

[21] Goddard M (2015). Competition in healthcare: good, bad or ugly?. Int J Health Policy Manag.

[22] Mwachofi A, Al-Assaf AF (2011). Health care market deviations from the ideal market. Sultan Qaboos Univ Med J.

[23] Forbes 2000 list.

[24] CBinsights.

[25] Johnson M, Lafley A (2010). Seizing the White Space: Business Model Innovation for Growth and Renewal.

[26] Johnson MW, Christensen CM, Kagermann H (2008). Reinventing your business model. Harvard Bus Rev.

