# Supplementary material for: Digital Transformation and Disruption of the Health Care Sector: Internet-Based Observational Study
Source: J Med Internet Res. 2018 Mar 27;20(3):e104. doi: 10.2196/jmir.9498 (PMC5893888; doi:10.2196/jmir.9498)
Supplement: Multimedia Appendix 1 [file jmir_v20i3e104_app1.pdf]

Start-up Comp. (with rank in Cbinsights Digital Health Deals)	Activities
Fitbit	www.fitbit.com
Ability Network	www.abilitynetwork.com
Privia Health	www.priviahealth.com
Oscar Health Insurence	www.hioscar.com
Guahao Technology	www.guahao.com
Change Healthcare	www.changehealthcare.com
Jawbone	www.jawbone.com
Veeva systems	www.veeva.com
Netsmart Technologies	www.ntst.com
NantHealth	www.nanthealth.com
Evolent Health	www.evolenthealth.com
Spring Rain Software	Link to explanation
Castlight Health	www.castlighthealth.com
Flatiron Health	www.flatiron.com
Clover Health	www.cloverhealth.com
TelaDoc	www.teladoc.com
DedalusThe Gym Group	www.thegymgroup.com
ZocDoc	www.zocdoc.com
Flatioron Health	www.flatiron.com
Alignment Healthcare	www.alignmenthealthcare.com
In Vitae	www.invitae.com
Proteus Digital Helath	www.proteus.com
XIFIN	www.xifin.com
23 and me	www.23andme.com
Butterfly Network	www.butterflynetinc.com
Helix	www.helix.com
Guahao Technology	www.guahao.com
Nantomics	www.nantomics.com
Evolent Health	www.evolenthealth.com
Healthline Media	www.healthline.com
Antenna79	www.antenna79.com
Virgin Pulse	www.virginpulse.com
Crossover Health	www.crossoverhealth.com
Care.com	www.care.com
Everbridge	www.everbridge.com
Drugstore.com	www.drugstore.com
Practo Technologies	www.practo.com
Medidata Solutions	www.mdsol.com
Clover Health	www.cloverhealth.com
Epocrates	www.epocrates.com
American Well	www.americanwell.com
Collective Health	www.collectivehealth.com
Bright Health	www.brighthealthplan.com

Homecare Homebase	www.hchb.com
Peloton Interactive	www.pelotoncycle.com
ZocDoc	www.zocdoc.com
Imprivata	www.imprivata.com
Practice Fusion	www.practicefusion.com/
Heath Catalyst	www.healthcatalyst.com
One Medical Group	www.onemedical.com
HeartFlow	www.heartflow.com
Cariomems	www.cardiomems.com
Castlight Health	www.castlighthealth.com
Clearcare	www.clearcareonline.com
PlanetRX	www.planetrx.com
Maestro Health Tech	www.maestrohealth.com
Dexcom	www.dexcom.com
Medsite	www.medsite.com
Kareo	www.kareo.com
Accoulade	www.accolade.com
Trubine	www.turbine.ai/
Vitalsmith	www.vitalsmith.com
Xbird	www.xbird.io
Medikeep	www.medikeep.eu/
Sendinaden	www.sendinaden.com/
Vitameter	www.vitameter.org
Viomedo	www.viomedo.de
Cortrium	www.cortrium.com
Fibricheck	www.fibricheck.com
Parica	www.parica.eu/
Pager	www.pager.com
Hometeamcare	www.hometeamcare.com
Medwand	www.medwand.com
Welldoc	www.welldoc.com
Rewalk	www.rewalk.com/de/
Telesofia	www.telesofia.org
Surgical Theater	www.surgicaltheater.net/
Totally Pregnant	www.iamtotally.com
Patient know best	www.patientsknowbest.com
iCouch	https://pro.icouch.me/
Adheretech	www.adheretech.com/
Biovotion	http://www.biovotion.com/
Infraredx	http://www.infraredx.com/
iRythmtech	http://www.irhythmtech.com/
Acutusmedical	https://acutusmedical.com/
Silk Road Medical	http://silkroadmed.com/
Cardiokinetix	http://www.cardiokinetix.com/de/
Cvrx	http://www.cvrx.com/
Ebr Systems	http://www.ebrsystemsinc.com/

Cardiofocus	http://www.cardiofocus.com/
Vytron	http://www.vytronus.com/
Jenavalve	http://www.jenavalve.de/
Mitralign	http://www.mitralign.com/
Direct flow medical	http://directflowmedical.com/
Cardiac dimensions	www.cardiacdimensions.com
Arstasis	http://www.arstasis.com/
Cardivamedical	http://www.cardivamedical.com/
Lombard Medical	http://www.lombardmedical.com/
Cardiovascular Systems	http://www.csi360.com/
Cellaegis	http://www.cellaegisdevices.com/
**Technology Comp. (with rank in Forbes2000)**	**Activities**
Apple	Apple Care kit, Platform for Apps
Apple	Apple acquired Glimpse (EMR)
AT&T	Connectivity (e.g. to the cloud, via VPN…)
AT&T	Collaboration with IBM to enhance Watson, a IoT-based platform
Verizon	Connectivity (similar to AT&T) as a service
Samsung	Platform offering for in-house and at home healthcare provision with a number of devices that resemble Apple to some extend
Microsoft	Solutions around patient engagement (e.g. follow up after in-house stay), better care continuum and care effectiveness
Microsoft	Analytical capabilities
Microsoft	Microsoft Corp. and TracFone Wireless announced collaboration to bring the benefits of smartphone technology and services to underserved and high-risk population (please enter link from comment by hand)
Alphabet	Calico
Alphabet	Baseline Joint Venture
Alphabet	Novartis contact lens Joint Venture (Diabetes)
Alphabet	GSK Joint Venture Galvani Bioelectronics
Alphabet	Sanofi Joint Venture Onduo (Diabetis management platform)
Alphabet	J&J Surgical robotics
Alphabet	Dexcom --> Mini glucose sensor
Alphabet	3M Health Information Systems --> population health measurement technology for managing clinical and financial performance.
Alphabet	Patient Management Ecosystem (e.g. in collaboration with healthcare provider [e.h. Harvard Univ.] & pharmacy)
Comcast	Similar to AT&T connectivity solutions
IBM	Watson: "The future of health is cognitive" --> Cloud data base combined with semantics focused AI-system
Siemens	Siemens Healthineers work on Hospital IT incl. Decision support systems
Siemens	Siemens Healthineers and IBM collaborate on population health measurement technology for managing clinical and financial performance
Intel	SaaS-solutions e.g. in Oncology
Cisco Systems	Provider for connected health data flow, IoT-tracking, work flow optimazation
GE	E Health Cloud --> largest application ecosystem for the healthcare industry; e.g. EMR: Get Real Health’s InstantPHR®

GE	GE Healthcare and Mission Health Launch First of a Kind Outcomes-Based Innovation Collaboration
GE	collaboration with Uppsala Bio (Process Optimization in the production of Biopharmaceuticals)
Deutsche Telekom	Hospital information systems
Deutsche Telekom	Trial connect service
Deutsche Telekom	eHealth platform
Deutsche Telekom	Ambient assisted living (e.g. smart home)
Oracle	Data warehouse and analytical solutions for healthcare providers
Oracle	Clinical trials software solutions
Oracle	Middleware to collect & analyse data
Telefonica	Digital Hospital incl. Imaging data warehouse, telehealth, teleadvice, M2M
Telefonica	Remote Patient Monitoring
Telefonica	Telefonica acquired controlling share in the Brazilian chronic care management provider Axismed
Walgreens Boots Alliance	Alliance with Prime Deal (pharmacy benefit manager)
Walgreens Boots Alliance	Alliance with OptumRx (pharmacy benefit manager)
Walgreens Boots Alliance	Collaboration with Qualcomm
Walgreens Boots Alliance	Alliance with HealthPrize Technologies (patient engagement platform)
Walgreens Boots Alliance	Boots MediCity Collaborator in Nottingham/UK
Medtronic	Medtronic and Fitbit
Medtronic	Collaboration with IBM Watson
Medtronic	Collaboration with Sanofi
Medtronic	Collaboration with Qualcomm
Medtronic	Collaboration with Samsung & Glooko around Diabetes Partnerships
Medtronic	Collaboration with Canary Health
Orange	Connecting patients and providers, some smaller experiments (Please enter by hand)
Honeywell	Connected health platform
Lookheed Martin	Healthcare Technology Alliance
Express Script	Cooperation with CWS around value based reimbursement
Express Script	Digital Care solutions through smart bottles
UPS	Hospital logistics
Time Warner	Connectivity (broad band cabel)
Hewlett Packard Enterprise	Focus on hardware with limited SaaS
Qualcomm	Medical grade IoT-connectivity
Qualcomm	Collaboration with Novartis (Qualcomm Life's 2net™ Platform for wireless data); HF-platform
Qualcomm	Collaboration with Boehringer Ingelheim (smart inhaler)
Qualcomm	Chronic diseases management programs
Qualcomm	Collaboration with Cerner
Qualcomm	Collaboration with Medtronic
Alibaba	online pharmacy
Hitachi	Hardware in the focus with added SaaS-solutions. Furthermore the company seems to prepare itself for ist next step in healtcare (e.g. collaboration with Quiagen)

SAP	SAP applies SaaS-concepts that worked in other industries to healthcare
SAP	collaboration with ASCO
Sony	Hardware (e.g. OR video equipment)
3M	Hospital IT
3M	Collaboration with Verily on population health measurementhealth
Tencent Holdings	Connectivity between care givers
BT Group	Focus on Connectivity and cloud based IT-solutions with limited activities on leveraging data to keep chronic ill patients out of the hospital
Danaher	Umbrella company that holds several life science companies (e.g. Leica)
China Unicom	Several local initiatives offering cloud based services to certain regions and cities
EMC	EMR- and other data bases with search capabilities
EMC	collaboration with eHealth-initiative in Saskatchewan
Amazon	Amazon Web Services (AWS) provide storage and analytical capabilities for hospitals, pharma & device comp.,
Amazon	Collaboration with Philips
Amazon	Collaboration with AHA
Fresenius	Dialysis-care, medications, Helios, Consultancy
Fresenius	Fresenius Ventures to invest in eHealth and other digital endevours
Panasonic	Hard- as well as software solutions to connect patients and physicians
Panasonic	Diabetes care business from Bayer
Schneider Electric	Healthcare & Hospital technology (e.g. combination of hardware and applicable software)
Telstra	Australian telecommunication company focused on Connectivity with patients incl. telehealth
Thermo Fisher Scientific	Along the lines of it lab equipment focus recent advances to illumine ("precision medicine moonshot")
Accenture	Accenture supports healthcare companies with a number of services that focus on productivity, change management, digital transformation etc.. A number of publication focus on changing business models in the life science sector towards value based reimbursement
Ericcson	Ericsson eHealth Croatia
Ericsson	Ericsson Mobile Health Remote Patient Monitoring
Baidu	Baidus AI Medical Assistant
Nokia	Nokia Healthcare
Nokia	Nokia Connected Health Withings
Tata Consultancy Services	TCS Diabetes Readmission Predictive Analytics Model
Tata Consultancy Services	Mad Mantra
Cognizant Technology	Smart Solutions for Government and Public Health Programs
RELX Group	Elsevier Patient engagment
Capgemini	Bookplan
Infosys	Healthcare
Infosys	Patient Relationship Managment Tool
Infosys	iHealth Analytics
Infosys	Pay for Perfomence

Fujitsu	Healthcare Solutions
VMware	CPOE System
VMware	Patient Engagment
VMware	Value Based Care
Wipro	360 Digital Patient Centricity suite & Other
ZTE	Healthcare Informatization Solution
ZTE	eHealth
ZTE	Cooperation with City of Düsseldorf
Adobe Systems	Healthcare
Adobe Systems	Boehringer Ingelheim Cooperation
Lenovo Group	Lenovo Solutions for Health
Lenovo Group	Patient-ID
Quanta Computer	www.quoca.net
Quanta Computer	Mobile Dialysis
HCL Technologies	Healthcare and Lifescience
CGI Group	Community Cae 360
Fiserv	Health Care Paying System
ATOS	Healthcare Services
Salesforce.com	Health Cloud , Collaboration with Merck Darmstadt
Salesforce.com	Stanley HealthCar, Philips collaboration, Similar to SAP
Symantec	Healthcare Solutions
Asustek Computer	Healthcare
Motorola Solutions	Communication Solutions
Intuit	Health Patient Portal
CA	Health Care Solutions
Check Point Software	Data security
Samsung SDS	Healthcare general
Samsung SDS	Smart Patient Engagment
Samsung SDS	Metronic and Samsung
Samsung SDS	Bioepis
Dassault Systemes	Variuos software products
Juniper Networks	Networks designed for Helathcare
Harris	NantHealth Acquires Harris Healthcare Solutions
Harris	Cooperation of Harris Health
Compal Electronics	New big Helathcare Unit
Citrix Systems	Hospital Software
VeriSign	Partnership with MS Healthvault
VeriSign	Partnership with Presideo Transitions and Organisations
Amdocs	Remote Monitoring Cloud based Solutions
Inventec	Joint venture with Advantec
Red Hat	Open Source Software
Autodesk	Hospital Planning
Palo Alto Networks,	Data Safety
**Lifescience Comp. (with rank in Frobes Top 2000)**	Activities
Allianz	Berlin-based insurtech startup distributes Allianz products in Europe.

Allianz	Insurer Blockchain Initiative (Blockchain Insurance Industry Initiative B3i)
Allianz	Dr. Allianz (Telemedicine)
Allianz	My health app Erstattungsanträge
AXA Group	Axa Selbstmanagment Portale
AXA Group	AXA Strategic Ventures leads $2 mln funding in healthcare app Wellth
AXA Group	The AXA PPP Health Tech & You programme
Johnson & Johnson	Cooperation with IBM Watson
Nestle	Nestle and Smasung cooperation healath care
UnitedHealth Group	United scoops up patient engagement platform
UnitedHealth Group	UnitedHealthcare and Konami Develop Innovative Program to Reduce Childhood Obesity with the Launch of "DanceDanceRevolution Classroom Edition"
UnitedHealth Group	Patient engagment
Pfizer	Patient engagment app
Pfizer	Cisco's visualization in Pfizer DD
Pfizer	Pfizer and IBM Watson
Novartis	SibA App for Comlience
Novartis	Aktiv bei Burstkrebs App
Novartis	Novartis Prävention App
Novartis	ViaOpta
Novartis	Qualcom & Novartis
Novartis	Novartis and Proteus for digital trails
Novartis	Novartis Launches the First Apple Watch App for the Visually Impaired
Novartis	Novartis and google Contact Lense
Novartis	Entresto Pay for Performence
Metlife	Metlfife Lumen Lab new Buisness Models
Siemens	Siemens and IBM Watson (more under tech)
CVS Health	CVS Health launches five new digital health features, including an Apple Watch app
CVS Health	CVS Health Introduces New Digital Pharmacy Tools to Help Make Medication Adherence Easier and More Convenient
CVS Health	CVS and IBM Wastson
Roche	Roche and Qualcomm Collaborate to Innovate Remote Patient Monitoring
Roche	Innovative Pricing Solutions
Sanofi	Sanofi value based pricing
Sanofi	Sanofi and google
Merck	Merck Pharma and Atena Value Based Care Contract
Bayer	IBM Watson and Bayer
GSK	Nanologica collaboration with GSK
GSK	GSK verlily GSK and Verily to establish Galvani Bioelectronics – a new company dedicated to the development of bioelectronic medicines
GSK	GSK Qualcom
GSK	GSK enters into partnership with Propeller Health to develop digital sensors for inhalers

GSK	Big pharma giant GSK says it is the first company in the arena to use Apple ResearchKit for real-world evidence.
MunichRE	HSB acquires Meshify tech startup Cloud-based IoT software connects commercial and industrial equipment
Walgreens Boot Alliance	Qualcomm announces new connected health collaboration with Walgreens
Walgreens Boot Alliance	Walgreens Goes Live with Data-Sharing Program for Suppliers Using Demand Signal System from Agentrics and Retail Solutions
Walgreens Boot Alliance	HealthPrize Technologies' Digital Patient Engagement, Education and Medication Adherence Platform Offered in Collaboration with Walgreens
Walgreens Boot Alliance	OptumRx and Walgreens Partner to Improve Consumer Convenience, Cost Savings and Outcomes
Walgreens Boot Alliance	Why the Walgreens/Prime Deal Could Transform the PBM Industry
Walgreens Boot Alliance	Walgreens Adds Teledermatology to its Digital Health Platform
Walgreens Boot Alliance	The Association between Participation in a Community Pharmacy's Digital Health Program and Flu Vaccination Rates
Walgreens Boot Alliance	The Association between Use of a Community Pharmacy's Mobile Pill Reminder App and Medication Adherence
Gilead Sciences	Gilead Sciences, Mobiquity make significant healthcare investment in London
Swiss Re	Series A funding of Biovotion (11 Mio in 2016)
Swiss Re	Insurance is "probing the water", e.g. organizing a conference on eHealth (http://media.cgd.swissre.com/documents/TransformingHealthcareTelemedicine_ConfReport_Feb2015.pdf)
Medtronic	Medtronic and Qualcomm Partner To Develop Fully Disposable Professional<br>CGM for Type 2 Diabetes - See more at: https://diatribe.org/medtronic-and-qualcomm-partner-develop-fully-disposable-professional-cgm-type-2-diabetes#sthash.OrlqG50Q.dpuf
Medtronic	Letter to senate
Medtronic	Value-Based Healthcare: A New Approach to Diabetes Care
Medtronic	MEDTRONIC AND HARVARD BUSINESS REVIEW
Medtronic	IBM Watson Health and Medtronic: Where devices, data, and patient<br>engagement meet
Medtronic	Medtronic launches connected app for pacemaker patients, but patients can't see the data
Medtronic	Increasing Patient Engagement through Effective Treatment Decision Support
Medtronic	Medtronic CareLink Network For Cardiac Device Patients
Amgen	Cigna inks results-based deals on pricey Amgen, Sanofi PCSK9 meds
Amgen	Harvard Pilgrim cements risk-based contract for pricey cholesterol drug Repatha
Amgen	Amgen uses algorithms to venture into digital healthcar
Allergan	Allergan Partners with SonarMD® to Develop Innovative IBS-D Patient<br>Engagement Platform for Better Identification and Care of IBS-D Patients
Anthem	New Anthem pay-for-performance program gives docs $3.1M
Anthem	Promoting Value-Based Contracting Arrangements with Eli Lilly

Anthem	Anthem Blue Cross' $38 Billion Move From Fee-For-Service Medicine
Anthem	Anthem unveils a slew of new digital healthcare tools
AbbVie	A number of regional digital programs accross Europe
McKesson	McKesson and TriZetto to Integrate ClaimsXten and QNXT Systems
McKesson	Equipping Care Managers for Success
McKesson	Prepping Oncology Practices for Value-Based Reimbursement
McKesson	Electronic Health Records and Practice Management Software Solutions
Aetna	How Aetna is using big data to improve patient health
Aetna	Cigna, Aetna enter outcomes-based contract with Novartis for heart drug
Aetna	Aetna and Apple collaborate on digital health and wellness for employers
Aetna	What Aetna's $37B acquisition of Humana could mean for digital health
AstraZeneca	AstraZeneca will pay for patients to have a personal digital health coach
AstraZeneca	Vida Health and AstraZeneca launch new app for post-heart attack recovery
AstraZeneca	AstraZeneca Drives Innovation with Intel Capital
AstraZeneca	AstraZeneca plans new connected inhaler trial to improve medication adherence in COPD patients
AstraZeneca	How AstraZeneca is Using Digital to Solve Pharma’s Biggest Challenges
AstraZeneca	AstraZeneca forms 'smart inhaler' digital health partnership
Cigna	Cigna wants more value-based reimbursements for systems treating at-risk patients
Cigna	Cigna inks results-based deals on pricey Amgen, Sanofi PCSK9 meds
Cigna	Cigna bets big on telehealth, Aetna on Apple Watch
Cigna	Cigna launches gamified digital health coaching
Cigna	CIGNA-Merck Outcomes Contract Hailed as ‘First Step,’ but Some Want More Data
Kroger	Kroger plans to buy digital health retailer for $280M
Abbott	Abbott EPD: the first digital marketing only launch in pharma
Teva Pharmaceutical	Teva Pharmaceuticals and IBM Partner to Build Global e-Health Solutions on the IBM Watson Health Cloud
Teva Pharmaceutical	Teva and Microchips Biotech Announce Partnership to Enhance Patient Outcomes through Digital Drug Delivery Technology
Eli Lilly	Patient Engagement App Challenge
Eli Lilly	Value-Based Pricing
Eli Lilly	Lilly & Anthem Find Common Ground in Value-Based Pricing
Fresenius	Fresenius and Kaiser Permanente Diabetes
Hospital Coporation America	HCA invests in cognitive computing technology Digital Reasoning
Hospital Coporation America	HCA invests in Sharecare for new patient engagement offering, innovation lab
Thermo Fisher	Thermo Fisher Scientific expands clinical trial services capabilities with new facility in India
Cardinal Health	Cardinal Health acquires TelePharm, a digital health startup aimed at retail pharmacies

Cardinal Health	Cardinal Health to Acquire OutcomesMTM
Cardinal Health	Cardinal Health To Acquire naviHealth, Leader In Post-Acute Care Management Solutions
Bristol Myers Squibb	Inovalon Announces Agreement with Bristol-Myers Squibb to Focus on<br>Real World Outcomes & Value-Based Contracting Initiatives
Air Liquide	https://www.airliquide.com/connected-innovation/ehealth-connected-solutions-effective-obstructive-sleep-apnea-treatment
Humana	Humana Partners With Pivotal to Rapidly Deliver Healthcare Solutions
Humana	Provider Medicare Quality P4P
Humana	Humana Spent $93M in Quality Payments for Provider Network
Humana	http://valuebasedcare.humana.com
Merck KGaA	eHealth solution for patients with MS (http://www.presseportal.de/pm/6873/3351175)
Novo Nordisk	Novo Nordisk, IBM Watson Health to Create 'Virtual Doctor'
Novo Nordisk	Patient Engagement with Novo Nordisk
Biogen	Biogen CEO says the biotech aims to develop wearable and ingestible devices
Celgene	Celgene, Novartis back precision medicine analytics startup after recent board addition from CMS
Stryker	http://www.prnewswire.com/news-releases/strykers-performance-solutions-announces-jointcoach-engagement-platform-designed-to-educate-and-navigate-patients-through-the-entire-episode-of-joint-replacement-care-300258556.html
Stryker	https://www.strykerperformancesolutions.com/solutions/alignment-strategies/transition-to-risk/bundled-payments
Stryker	https://www.strykerperformancesolutions.com/articles/stryker-explores-broader-risk-based-contracts
Colgate Palmolive	http://investor.colgate.com/releasedetail.cfm?ReleaseID=710979
Baxter	https://www.sciencedaily.com/releases/2016/06/160621144030.htm
BD	http://marketrealist.com/2016/06/how-becton-dickinson-is-driving-growth-through-collaborations/
Astellas Pharma	Humana and Astellas Form Research Collaboration to Improve Health Care Delivery for Seniors (http://cts.businesswire.com/ct/CT?id=smartlink&url=http://www.humana.com&esheet=50568707&lan=en-US&anchor=Humana+Inc.&index=1&md5=3a9dde39caa650820497a1bc315f92ab)
Mylan	Minor patient adherence platform in Canada (http://www.pharmafile.com/news/501512/mylan-launches-digital-adherence-programme-heart-patients)
Mylan	Minor activities in neurological disorders (http://scharr-rat.blogspot.de/2015/06/tim-telehealth-in-mnd-study.html)
Otsuka Holding	Joint venture (Otsuka Digital Health) with IBM in psychiatric disorder (http://www.pmlive.com/blogs/digital_intelligence/archive/2016/june/otsuka_joins_forces_with_ibm_for_watson-based_digital_health_venture_1041155?SQ_DESIGN_NAME=2)
Otsuka Holding	Proteus & Otsuka submit first commercial drug with built-in sensor to FDA in 2015 (http://www.mobihealthnews.com/46680/proteus-otsuka-submit-first-commercial-drug-with-built-in-sensor-to-fda)
Daiichi Sanyko	Telehealth initiative in the US on engaging with patients that have Afib together with Harvard's Center of Connected Health

	(http://connectedhealth.partners.org/news-and-events/media-center/announcements/daiichi-sankyo-mobile-app.aspx) (http://www.mobihealthnews.com/40991/why-partners-and-daiichi-sankyo-partnered-on-an-afib-remote-patient-monitoring-pilot)
Daiichi Sanyko	Minor initiative in the UK on engaging with patients that have Afib (http://www.pmlive.com/blogs/digital_intelligence/archive/2016/february/daiichi_sankyo_collaborates_on_atrial_fibrillation_resource_for_e-patients_935610)
Shire	Minor activities around ehealth enhanced drug trials (http://www.mobihealthnews.com/33124/shire-drug-trials-should-be-marketed-as-a-healthcare-service)
Takeda Pharmaceutical	Company wide initiative (Takeda Digital Accelerator) to explore "digital" technology along their value chain (http://www.fiercepharma.com/marketing/getting-serious-about-digital-takeda-walks-walk-its-digital-accelerator-model)
Baxalta (2015 acquired by Shire)	2015 publication on a reimbursement-strategy in hemophilia (http://www.tandfonline.com/doi/pdf/10.1080/14737167.2016.1178066?needAccess=true)
Reinsurance Group of America	Minor investment in APP producer (UK) roadtohealth (http://www.mobihealthnews.com/content/health-risk-assessment-app-maker-roadtohealth-gets-4m-reinsurance-group-america)
Reinsurance Group of America	Interesting position paper on insurance's homepage around Patient Generated Personal Health (PGPH) data (http://www.rgare.com/knowledge-center/media/articles/ramifications-of-utility-importance-gains-for-patient-generated-health-data-(pghd))
Sinopharm Group	China National Pharmaceutical Group Corp. (CNPGC) known as Sinopharm is a Chinese state-owned enterprise. The corporation was the indirect major shareholder of publicly traded companies Sinopharm Group. The company appears to engage on different fronts into eHealth but links are difficult to understand (e.g. one project with IBM)
Valeant Pharmaceuticals	App-dev together with IBM for eye surgeons (http://www.businessfinancenews.com/28874-valeant-pharmaceuticals-intl-inc-vrx-entering-new-era-of-medical-applicatio/)
Valeant Pharmaceuticals	Valeant received neg. press in 2016 for dramatic price increases for single source off-patent CV-drugs (http://ashp.org/menu/News/PharmacyNews/NewsArticle.aspx?id=4309)
Mitsubishi Chemical	Mitsubishi Tanabe Pharma (http://www.mt-pharma.co.jp/e/develop/pipeline/e_pipeline1609.pdf) and the Lifescience Inst. (http://www.lsii.co.jp/en/) are main activities in healthcare sector. NO major eHealth initiatives
Insurance Australia Group	Health insurance that has interests in the digital transformation of its sector (http://svicenter.com/my-product/iag-australia/) but no initiatives found in public domaine
DaVita	DaVita Inc. is one of the largest kidney care companies in the United States. 2015 a collaboration with Qualcomm (https://www.qualcomm.com/news/releases/2015/08/31/qualcomm-life-announces-new-connected-health-collaborations-connect-2015) on chronic care management programs was announced.
DaVita	2014: Collaboration with Medtronic to understand CV-morbidity in CKD patients better (http://www.news-medical.net/news/20141116/DaVita-Kidney-Care-Medtronic-partner-to-better-understand-cardiovascular-health-in-ESRD-patients.aspx)

DaVita	Nov 2016: DaVita partners with Vsee to use video technology to engage with patients in the home setting (http://www.4-traders.com/DAVITA-INC-12378/news/Davita-Get-Real-Health-s-New-Partnership-with-VSee-Enhances-Patient-Engagement-with-Real-time-Vide-23473068/)
Beiersdorf	N/A (new platform to interact with external R&D-comp. --> Innoget (http://uk.fashionnetwork.com/news/Beiersdorf-boosts-collaboration-on-innovation-with-Innoget,727665.html#.WGN2-VXhDIU))
St Jude Medical	Acquired by Abbott in 2016. In HF e.g. CardioMems system launched which faces reimbursement challenges despite being superior to SoC (http://media.sjm.com/newsroom/media-kits/heart-failure-hypertension/united-states/default.aspx) and (http://www.mddionline.com/blog/devicetalk/was-medtronic-right-about-st-jude-medicals-cardiomems)
St Jude Medical	http://www.mddionline.com/blog/devicetalk/facing-reimbursement-hurdles-st-jude-seek-cms-national-coverage-1-28-15)
LabCorp	One of the world largest lab testing companies that acquired Covance (2015) and made advances at another CRO (INC) in 2016. In a recent initiative the companies tries to "cut the middleman out" in it lab-value offerings directed at consumers without physician's interface (https://www.ehidc.org/resources/498-labcorp)
LabCorp	2014: Funding for Vivify (http://www.businesswire.com/news/home/20141125005774/en/Vivify-Health-Secures-Funding-Global-Strategic-Partners)
Universal Health Services, Inc.	UHS is one of the largest hospital management companies in the United States.... its subsidiaries 24 inpatient acute care hospitals, 3 free-standing emergency departments and 213 inpatient and 16 outpatient behavioral health care facilities located in 37 states, Washington, D.C., the United Kingdom, Puerto Rico and the U.S. Virgin Islands. >115 telehealth projects ongoing focused on lowering readmissions, cutting unnecessary emergency department use, and improving outcomes and patient satisfaction (http://www.uhsinc.com/behavioral-health/behavioral-health-integration-solutions/)
Boston Scientific	US based device company that offers hardware solutions for patient remote monitoring (http://www.bostonscientific.com/en-US/products/remote-patient-monitoring/preventice-bodyguardian.html) and engages in numerous collaborations (incl. Karolinska Hospital) to fully leverage the digital transformation of the healtcare sector (http://www.bostonscientific.com/en-US/products/remote-patient-monitoring.html) and (http://www.karolinska.se/en/karolinska-university-hospital/Innovation/innovation-partnership/boston-scientific/)
Boston Scientific	Collaboration with consultancies around value-based-care (http://ehealth.eletsonline.com/2015/05/boston-scientific-collaborates-medaxiom-togethermd/) and (http://news.bostonscientific.com/2016-01-28-Boston-Scientific-and-Accenture-Develop-Data-Driven-Digital-Health-Solution-to-Help-Improve-Patient-Outcomes-and-Reduce-Cost-of-Treating-Chronic-Cardiovascular-Conditions)
Boston Scientific	Consultancy around ist products with focus on value-based care (http://www.bostonscientific.com/en-EU/advantics.html)
Zimmer Biomet	Medical device company focused on orthopedic devices...spun off from Bristol-Myers Squibb. Digital activities incl. home based physiotherapy (http://www.mobihealthnews.com/content/zimmer-

	biomet-buys-home-based-physical-therapy-company-respondwell)
Zimmer Biomet	Value based care in the focus (http://www.prnewswire.com/news-releases/zimmer-biomet-unveils-signature-solutions-300303747.html) focus on • Interactive patient engagement tools for patient education, communication and adherence to protocols • Data mining and analysis platform to collect patient-reported outcomes • Consulting services • Digital health software solutions • Personalized, patient-specific medical technologies and instruments to improve quality and outcomes as well as streamline workflow
Onex Corpoation	N/A (Onex Corporation is a private equity investment firm and holding company based out of Toronto, Ontario. As of 2016, the firm has over $22 billion of assets under management.Investments in a number of eHealth companies e.g. http://www.cliniconex.com/2016/10/cliniconex-expands-ottawa-team-armed-with-new-funding-led-by-capital-angel-network-and-wesley-clover/)
Henry Schein Inc.	Henry Schein Inc. is a distributor of health care products and services. Collaboration with digital healthcare solution provider Medpod (http://www.medpodinc.com/)
Henry Schein Inc.	Partner in the Quality payment program of the US-government with focus on diabetes, beta-blocker use after MI and hypertension (https://qpp.cms.gov/) and (https://www.henryschein.com/us-en/Medical/ResourceCenter/quality-value-based-care.aspx)
Henry Schein Inc.	Practise management platform (http://dentrix.com/about-us/about-dentrix)
Henry Schein Inc.	Diabetes management platform (https://www.henryschein.com/us-en/340B/ResourceCenter/comprehensive-diabetes-care.aspx)
Quest Diagnostics	MedPlus, the healthcare information technology subsidiary of Quest Diagnostics, is a leading developer and integrator of clinical connectivity and healthcare data management solutions (http://ir.questdiagnostics.com/phoenix.zhtml?c=82068&p=irol-newsArticl
Quest Diagnostics	Contrary to LabCorp Quest aims at the physician segment with its online platforms (http://itelemedicine.com/news/virtual-diagnostic-testing/)
Quest Diagnostics	Collaboration with IBM-Watson to offer lab/genetic and analytical capabilities to physicians (http://www.mobihealthnews.com/content/ibm-teams-quest-diagnostics-precision-medicine-service-and-7-more-digital-health-deals)
Centene	Centene Corporation is a leading multi-line healthcare enterprise that contracts with government healthcare programs, healthcare and commercial organizations to provide specialty services, including behavioral healthcare services through Cenpatico, care management software, correctional systems healthcare, in-home health services, life and health management, vision, pharmacy benefits management, specialty pharmacy and telehealth services (e.g. http://www.centene.com/programs/connections-plus/).
Regeneron Pharmaceuticals	Biotechnology company originally focused on neurotrophic factors and regenerative capabilities it branched out into cytokines and tyrosine kinase receptor inh. Several initiatives with Mt. Sinai, Mayo and Geisinger to analyse genomic data sets (https://www.genomeweb.com/sequencing/regeneron-launches-100k-patient-genomics-study-geisinger-forms-new-genetics-cent?utm_source=TrendMD&utm_medium=TrendMD&utm_campaign=1)

Sun Pharma Industries	Sun Pharmaceutical Industries is an Indian multinational pharmaceutical company that manufactures and sells pharmaceutical formulations and active pharmaceutical ingredients (APIs) primarily in India and the United States. Collaboration with gomohealth, a digital patient engagement company (https://gomohealth.com/)
Rite Aid (soon Walgreens (see above))	Rite Aid is the largest drugstore chain on the East Coast and the third largest in the U.S.. 2015 Walgreens offered >17 billion US $ to acquire it. Initiative to directly address patients through Mdlive (http://www.drugstorenews.com/article/walgreens-telehealth-expansion-other-pilots-shed-light-pharmacy%E2%80%99s-changing-role)
Community Health Systems	Community Health Systems Inc. is the largest provider of general hospital healthcare services in the United States. It cooperates with America Well on Telehealth services (https://www.americanwell.com/the-top-5-reasons-health-systems-like-chs-are-adopting-telehealth/)
Tenet Healthcare	Tenet Healthcare Corporation is a multinational investor-owned healthcare services that operates hospitals and acute care facilities (mainly in US). Its subsidary Conifer Health Solutions offers digital solutions to hospitals and practices (http://www.healthcareitnews.com/news/tenet-subsidiary-acquires-rcm-developer) and (http://coniferhealth.com/)
Intuitive Surgical	Intuitive produces surgical robotic solutions. Collaboration with intouch-telehealth provider (http://www.intouchhealth.com/) to Jointly Develop Patient Informatics Network and Cloud-based Operating Room Data Solutions
