# Supplementary material for: Digital Transformation and Disruption of the Health Care Sector: Internet-Based Observational Study
Source: J Med Internet Res. 2018 Mar 27;20(3):e104. doi: 10.2196/jmir.9498 (PMC5893888; doi:10.2196/jmir.9498)
Supplement: Multimedia Appendix 2 [file jmir_v20i3e104_app2.pdf]

Rank	Name	Costumer value proposition given 0 (not given) - 4 (fully visible)	Mean of Key Resources, Key Processes and Profit formula can be identified	Total Mean	Comment	Classification	Targets	Located
1a	Proteus Digital Health	3,67	3,00	3,33	Proteus Discover want to increase adherence via an ingestible sensor, sensor patch, mobile application and a provider porta	Device	Adherence	Redwood City, California, United States
2a	Jawbone	3,43	2,95	3,19	Fitness Tracker	Device	Lifestyle	San Francisco, California, United States
3a	Fitbit	3,29	2,90	3,10	Fitness Tracker	Device	Lifestyle	San Francisco, California, United States
4a	Dexcom	3,33	2,78	3,06	Continuous glucose monitoring via mobile application	Device	Adherence	San Diego, California, United States
5a	iRhythmtech	3,29	2,52	2,90	Cardiac arrhythmic monitor via a sensor patch and cloud based analytics	Device	Diagnostic	San Francisco, California, United States
6a	Telesofia Medical	3,20	2,53	2,87	Patient engagement and management platform	Platform	Patient engagement	Tel Aviv, Tel Aviv, Israel
7a	Privia Health	3,00	2,29	2,64	Platform to connect patients and doctors	Platform	Patient engagement	Arlington, Virginia, United States
8a	Vitalsmith	2,67	2,61	2,64	Ovulation testing via saliva and smartphone device	Service	Lifestyle	Gangneung, Kangwon-do, South Korea
9a	Adheretech	3,00	2,24	2,62	Wireless pill bottles to increase adherence	Device	Adherence	New York, New York, United States
10a	Oscar Health Insurance	2,71	2,48	2,60	Technology-centered insurance provider	Service	Prevention	New York, New York, United States

11 a	American Well	3,00	2,17	2,58	Telehealth technology to improve access to affordable care	Platform	Patient engage ment	Boston, Massachus etts, United States
12 a	Virgin Pulse	2,83	2,28	2,56	Develops technologies to improve employees engagement through healthier lifestyle	Softwar e	Preventi on	Framingha m, Massachus etts, United States
13 a	Crossover Health	2,83	2,22	2,53	App for simplifying appointments for health services	Platform	Lifestyle	Aliso Viejo, California, United States
14 a	Cardiomems	2,50	2,56	2,53	Heart failure management system	Device	Diagnos tic	Atlanta, Georgia, United States
15 a	Fibricheck	2,67	2,33	2,50	App to monitor your heart rhythm via smartphone	Softwar e	Diagnos tic	Hasselt, Limburg, Belgium
16 a	23 and me	2,50	2,44	2,47	Genetic testing to prevent diseases via kit that is mailed back from home	Service	Diagnos tic	Mountain View, California, United States
17 a	The Gym Group	2,50	2,39	2,44	Gym provider with more flexible offers all over the country and a new way to exercise	Service	Preventi on	Guildford, Surrey, United Kingdom
18 a	Vitameter	2,67	2,22	2,44	Vitamin testing & dosage via one drop of blood and a technical device	Device	Lifestyle	Kitchener, Ontario, Canada
19 a	Mitralign	2,60	2,27	2,43	Non-surgical mitral valve repair using latest catheter and imaging technology	Device	Treatme nt	Tewksbury, Massachus etts, United States
20 a	Xbird	2,57	2,29	2,43	Use machine learning, big data, sensor analyzing to detect critical health events prior to appearance	Service	Diagnos tic	Berlin, Berlin, Germany
1b	Medtronic	3,50	2,67	3,08	Partnership with fitbit to track glucose levels and physical activity data via an app	Softwar e	Preventi on	Minneapoli s, Minnesota, United States
2b	Samsung	3,43	2,57	3,00	Platform offering for in-house and at home health care provision with a number of devices for seniors	Platform	Preventi on	Suwon, Chung Chong-namdo, South Korea
3b	Microsoft	3,33	2,67	3,00	Health cloud	Platform	Quality	Redmond,

					information management system		Management	Washington, United States
4b	Alphabet	3,14	2,71	2,93	Autofocus contact lens to measure blood glucose levels or for people with farsightedness	Device	Adherence	Mountain View, California, United States
5b	Qualcomm	3,33	2,39	2,86	Wireless tracker for its respite inhalers	Device	Adherence	San Diego, California, United States
6b	Apple	3,14	2,52	2,83	Apple care kit: platform for Apps	Software	Quality Management	Cupertino, California, United States
7b	Alphabet	3,14	2,48	2,81	Partnership with Dexcom to develop smaller and better glucose sensors	Device	Adherence	Mountain View, California, United States
8b	Medtronic	3,17	2,28	2,72	Partnership with Sanofi with focus on developing devices and care-management services for chronical diseases	Device	Adherence	Minneapolis, Minnesota, United States
9b	Microsoft	3,00	2,44	2,72	Microsoft Cloud solutions to improve patient outcomes, lower costs, and increase efficiency	Software	Patient engagement	Redmond, Washington, United States
10b	Alphabet	3,00	2,22	2,61	Verily brings together technology and life sciences to explore new ways in health and diseases	Software	Quality Management	Mountain View, California, United States
11b	AT&T	2,86	2,33	2,60	Connectivity provider to track and monitor patients to improve outcome	Platform	Quality Management	Dallas, Texas, United States
12b	Qualcomm	3,17	2,00	2,58	Chronic diseases management programs	Platform	Quality Management	San Diego, California, United States
13b	Medtronic	3,00	2,17	2,58	Aim to Improve care and health outcomes for people with type 2 diabetes	Device	Adherence	Minneapolis, Minnesota, United States
14b	BM	2,83	2,33	2,58	IBM Watson cloud data base combined with semantics focused AI-system to deliver better health	Software	Diagnostic	Armonk, New York, United States

15 b	Alphabet	3,14	2,00	2,57	Joint Venture with Sanofi to develop diabetes management platform	Softwar e	Quality Manage ment	Mountain View, California, United States
16 b	Alphabet	3,00	2,11	2,56	Joint Venture with GSK to develop future generation continuous glucose monitoring systems to improve health outcomes	Device	Adhere nce	Mountain View, California, United States
17 b	Verizon	2,71	2,33	2,52	Connectivity provider to transform healthcare	Platform	Quality Manage ment	New York, New York, United States
18 b	Qualcomm	3,00	2,00	2,50	Medical grade Internet of Things-connectivity platform	Softwar e	Quality Manage ment	San Diego, California, United States
19 b	SAP	2,83	2,17	2,50	Providing in-memory data platform to improve patient treatment and change cancer care	Platform	Quality Manage ment	Walldorf, Baden-Wurttembe rg, Germany
20 b	Alphabet	2,71	2,19	2,45	Partnership with J&J to develop better surgical robotics	Device	Treatme nt	Mountain View, California, United States
1c	Novartis	3,14	2,71	2,93	Deal with payers that heart failure drug Entresto will be paid-for-performance	VBC	Quality Manage ment	Basel, Basel-Stadt, Switzerlan d
2c	GSK	3,40	2,33	2,87	Partnership with Propeller Health to develop digital sensors for inhaler to improve adherence and outcome	Device	Adhere nce	Middlesex, New Jersey, United States
3c	Roche	3,17	2,39	2,78	Partnership with Qualmcomm to innovate and improve remote monitoring and management of patients with chronical diseases	Platform	Quality Manage ment	Basel, Basel-Stadt, Switzerlan d
4c	St Jude Medical	3,00	2,53	2,77	Developing innovative solutions for heart failure disease management	Device	Treatme nt	Saint Paul, Minnesota, United States
5c	Sanofi	3,17	2,33	2,75	Development of	Device	Adhere	Paris, Ile-

					diameters devices		nce	de-France, France
6c	Baxalta (2015 acquired by Shire)	3,20	2,27	2,73	Developing innovative outcome based care model for hemophilia management	VBC	Quality Manage ment	Illinois City, Illinois, United States
7c	Merck KGaA	3,40	2,00	2,70	Partnership with HAPPYneuron for cognitive training program for multiple sclerosis patients via platforms	Softwar e	Quality Manage ment	Darmstadt, Hessen, Germany
8c	AstraZeneca	3,00	2,40	2,70	Connected inhaler trial to improve adherence in chronic obstructive pulmonary disease (COPD) patients	Device	Adhere nce	London, London, United Kingdom
9c	Novartis	3,00	2,39	2,69	Created app for visually impaired people to walk by foot	Softwar e	Treatme nt	Basel, Basel-Stadt, Switzerlan d
10 c	AstraZeneca	3,00	2,33	2,67	Partnership with Adherium to develop smart inhaler for patients with asthma	Device	Adhere nce	London, London, United Kingdom
11 c	Teva Pharmaceuti cal	3,00	2,28	2,64	Partnership with Microchips Biotech to develop digital drug delivery technology for improved patient outcome	Device	Treatme nt	Tiqva, HaMerkaz, Israel
12 c	Sanofi	2,83	2,44	2,64	Value-based pricing deal with insurer Cigna for new cholesterol drug	VBC	Quality Manage ment	Paris, Ile-de-France, France
13 c	Walgreens Boot Alliance	3,00	2,27	2,63	Mobile pill reminder app to increase adherence	Softwar e	Adhere nce	Deerfield, Illinois, United States
14 c	CVS Health	3,00	2,27	2,63	Developing digital tools to help customers manage their health in simpler ways	Softwar e	Quality Manage ment	Woonsock et, Rhode Island, United States
15 c	Merck	3,00	2,25	2,63	Value-based deal with Aetna for type 2 diabetes medications (Januvia and Janumet)	VBC	Quality Manage ment	Darmstadt, Hessen, Germany
16 c	Pfizer	3,00	2,17	2,58	Developing patient-centered mobile	Softwar e	Patient engage	New York, New York,

					health apps		ment	United States
17c	Novartis	3,00	2,17	2,58	Partnership with Qualcomm for digital inhaler device to empower patients with COPD to manage their disease	Device	Treatment	Basel, Basel-Stadt, Switzerland
18c	GSK	3,00	2,06	2,53	Patient-centered research study with use of the iPhone for rheumatoid arthritis, to improve knowledge of the disease	Software	Quality Management	Middlesex, New Jersey, United States
19c	Daiichi Sanyko	3,00	2,00	2,50	Using telehealth to engage patients with atrial fibrillation	Platform	Patient engagement	Chūō, Tokio, Japan
20c	CVS Health	2,83	2,17	2,50	Apple Watch app to inform customers when recipes are ready	Software	Adherence	Woonsocket, Rhode Island, United States
