# Supplementary figures and images for: Digital Transformation and Disruption of the Health Care Sector: Internet-Based Observational Study
Source: J Med Internet Res. 2018 Mar 27;20(3):e104. doi: 10.2196/jmir.9498 (PMC5893888; doi:10.2196/jmir.9498)

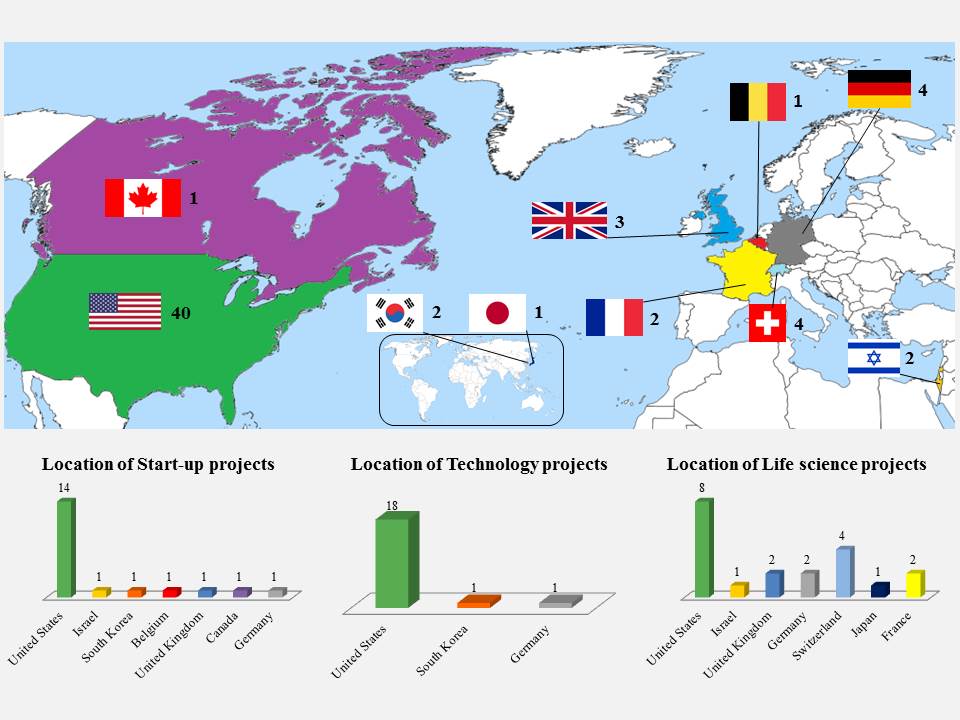

Supplement: Multimedia Appendix 3 [file jmir_v20i3e104_app3.jpg]

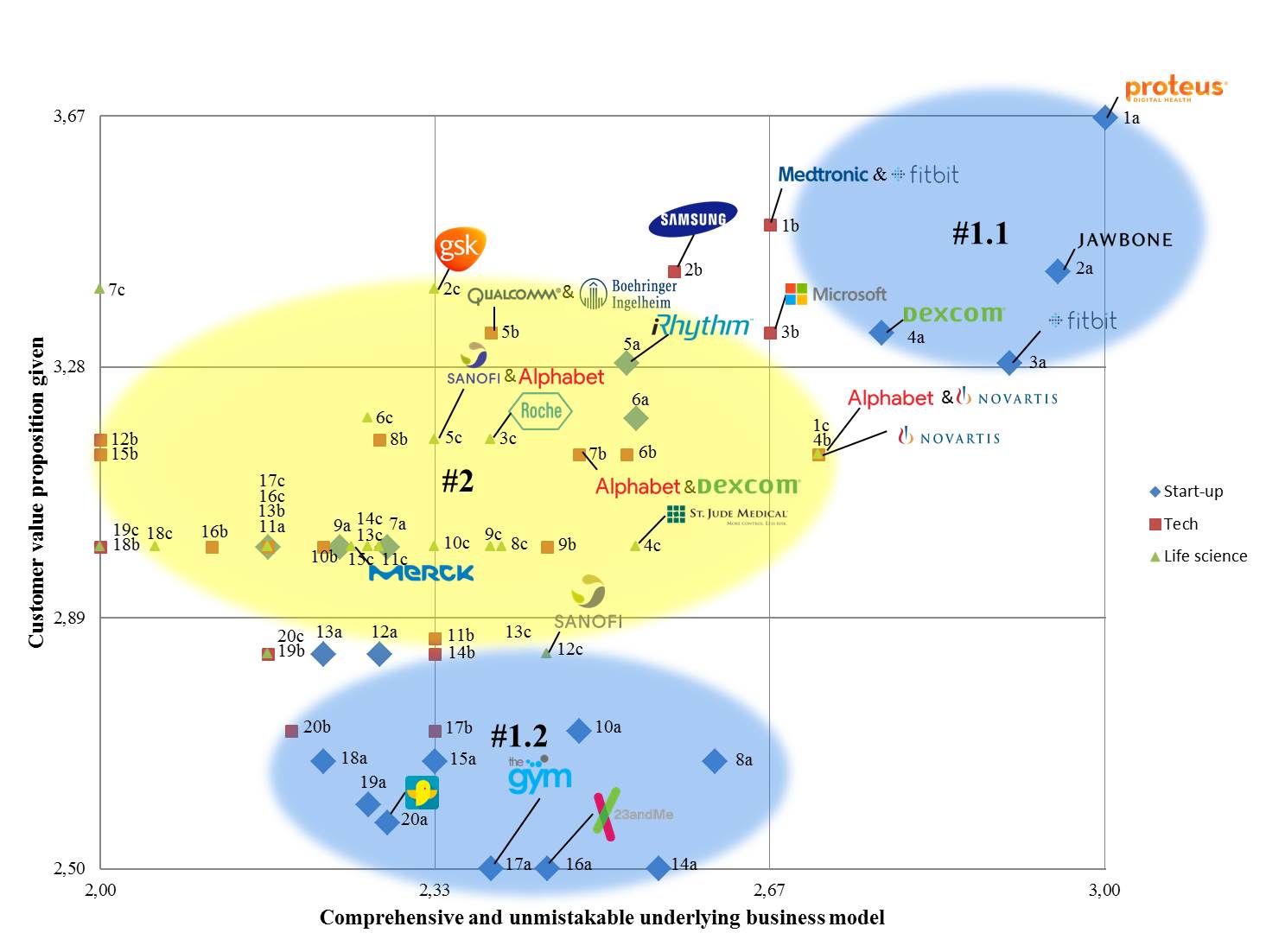

Supplement: Multimedia Appendix 4 [file jmir_v20i3e104_app4.jpg]
